# The Retrieval and Effect of Core Parameters for Near-Field Inter-Body Coupling Communication

**DOI:** 10.3390/s23125521

**Published:** 2023-06-12

**Authors:** Xu Zhang, Yong Song, Ya Zhou, Maoyuan Li, Wu Ren, Yizhu Ma, Changxiang Li, Yubo Cao

**Affiliations:** 1 School of Optics and Photonics, Beijing Institute of Technology, Beijing 100081, China; xuzhang_bit@bit.edu.cn (X.Z.); yzma@bit.edu.cn (Y.M.); changxiangli@bit.edu.cn (C.L.); yubo_cao@bit.edu.cn (Y.C.); 2 Department of Electronic Systems, Norwegian University of Science and Technology, 7491 Trondheim, Norway; maoyuan.li.bit@gmail.com; 3 The Intervention Centre, Oslo University Hospital, 0372 Oslo, Norway; 4 School of Integrated Circuits and Electronics, Beijing Institute of Technology, Beijing 100081, China; renwu@bit.edu.cn

**Keywords:** near-field inter-body coupling communication, channel model, channel characteristics

## Abstract

The potential of the Internet of Body (IoB) to support healthcare systems in the future lies in its ability to enable proactive wellness screening through the early detection and prevention of diseases. One promising technology for facilitating IoB applications is near-field inter-body coupling communication (NF-IBCC), which features lower power consumption and higher data security when compared to conventional radio frequency (RF) communication. However, designing efficient transceivers requires a profound understanding of the channel characteristics of NF-IBCC, which remain unclear due to significant differences in the magnitude and passband characteristics of existing research. In response to this problem, this paper clarifies the physical mechanisms of the differences in the magnitude and passband characteristics of NF-IBCC channel characteristics in existing research work through the core parameters that determine the gain of the NF-IBCC system. The core parameters of NF-IBCC are extracted through the combination of transfer functions, finite element simulations, and physical experiments. The core parameters include the inter-body coupling capacitance ($C_{H}$), the load impedance ($Z_{L}$), and the capacitance ($C_{air}$), coupled by two floating transceiver grounds. The results illustrate that $C_{H}$, and particularly $C_{air}$, primarily determine the gain magnitude. Moreover, $Z_{L}$ mainly determines the passband characteristics of the NF-IBCC system gain. Based on these findings, we propose a simplified equivalent circuit model containing only core parameters, which can accurately capture the gain characteristics of the NF-IBCC system and help to concisely describe the channel characteristics of the system. This work lays a theoretical foundation for developing efficient and reliable NF-IBCC systems that can support IoB for early disease detection and prevention in healthcare applications. The potential benefits of IoB and NF-IBCC technology can, thus, be fully realized by developing optimized transceiver designs based on a comprehensive understanding of the channel characteristics.

## 1. Introduction

Every year, millions of people across the globe die from chronic, infectious, and cancerous diseases, posing significant challenges to life expectancy [1,2]. Early screening and diagnosis are crucial in preventing and managing fatal or chronic ailments. The Internet of Body (IoB) represents a promising extension of the Internet of Things (IoT); it utilizes connected devices in, on, and around the human body to enable various applications [3,4,5]. The IoB offers proactive wellness screening and post-surgery or medication monitoring opportunities, among others [6,7].

The IoB for medical applications utilizes two types of information exchange modes, namely, in vivo and in vitro communication [8,9]. In vivo communication takes place through human tissues, serving as the primary channel for vital signals to interact wirelessly via implants [10,11]. In vitro communication involves information interaction between external hubs, which involves transmitting monitored vital signs from the hub to the doctor for information processing and diagnosis. Considering the doctor’s diagnosis, in vitro (non-contact) communication is preferred to avoid the potential risk of disease spread, with air serving as the primary communication medium. Moreover, it is imperative that the quality of service (QoS) of the IoB possesses relatively strict security and low consumption characteristics, given the sensitivity of vital signals [12].

**Figure 1:**
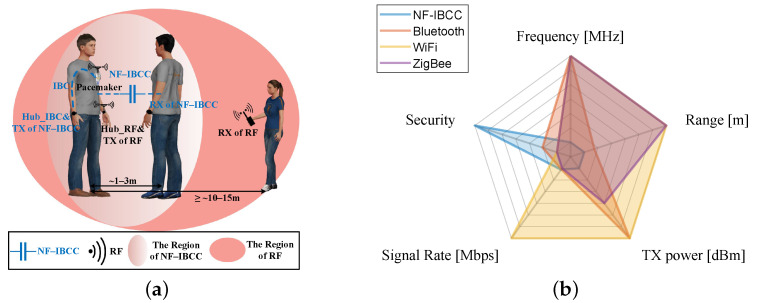
(**a**) Information interaction between the pacemaker and Hub_IBC can be realized through intra-body communication (IBC) while the information interaction between human bodies can be achieved through NF-IBCC. Comparing the effective distance of NF-IBCC with traditional RF communication technology, it is observed that NF-IBCC provides relatively higher information security. The human figures were created using the open-source software MakeHuman. (**b**) A comparison of NF-IBCC and traditional RF communication technologies, including Bluetooth, Wi-Fi, and ZigBee, was performed based on the operating frequency, communication range, transmission power at the TX end, information rate, and information security. It was found that NF-IBCC has low power consumption and high information security characteristics [6,19,20].

A strategy for balancing the trade-off between the risks of disease transmission and data security has been proposed, namely, near-field inter-body coupling communication (NF-IBCC) [13]. This strategy adopts the concept of near-field intra-body communication (NF-IBC), also referred to as human body communication (HBC), which provides advantages such as low power consumption and high data security. Specifically, the electromagnetic fields are distributed in the vicinity of the human body when the source TX electrodes are in contact with one human body. Strong coupling occurs without physical contact, when another body, equipped with RX, approaches the near-field region. As depicted in Figure 1a,b, the operating frequency of NF-IBCC is below 10 MHz [14]. In comparison to radio frequency (RF) communication technologies, such as Bluetooth, Wi-Fi, and ZigBee, the evanescent wave attenuates rapidly as the transmission distance increases. For transmitted power levels less than 1 mW, the near-field coupling distance is less than 3 m [15]. Given that the ratio of the transmission distance to the operating wavelength is substantially less than 0.1, it is anticipated that radiation levels will be lower, thereby enhancing the security of the physical layer [16].

A profound understanding of the channel characteristics of NF-IBCC is necessary for designing efficient transceivers [17]. However, some works reported inconsistent results in terms of the passband nature and its gain magnitude, which can be attributed to different experimental equipment. For instance, in [18], the channel characteristics were ascertained by a vector network analyzer (VNA). The measurements employed a balun to eliminate the common ground effect, and the channel exhibited high-pass characteristics, with the channel gain magnitude varying between −48 and −22 dB. In [14], the authors obtained the channel characteristics by using discrete instruments, including handheld signal generators and receivers. The high-input capacitance ($Z_{L}=\frac{1}{sC_{L}}$) was utilized on the RX side, and the channel exhibited a flat band with a gain magnitude of −70 dB. In [13], the channel characteristics at a distance of 0.5 m were obtained by a handheld signal generator and a spectrum analyzer. The high-pass characteristics with a gain magnitude between −60 and −40 dB were found. In essence, the channel gain of NF-IBCC is greatly influenced by the grounding conditions and load. In practical applications, it is crucial to ensure that the measuring instruments are sufficiently grounded to obtain precise channel characteristics, given that the transceiver’s ground is completely isolated. Additionally, the passband nature of the channel may differ based on the load impedance of the transceiver. These factors contribute to the variability of the channel characteristics, rendering them unreproducible, and hampering the development of effective transceivers. Therefore, further research is imperative to investigate the effects of these components on channel characteristics and establish repeatable channel models for NF-IBCC.

In this paper, our main focus is to investigate the factors responsible for the unrepeatable channel characteristics of NF-IBCC and their impact on the overall channel behavior. Specifically, we extracted the core parameters that determine the gain and passband of the NF-IBCC system through circuit analysis, finite element simulation, and physical experiments. The core parameters include the inter-body coupling capacitance ($C_{H}$), the load impedance ($Z_{L}$), and the capacitance ($C_{air}$), coupled by two floating transceiver grounds. The results illustrate that $C_{H}$, and particularly $C_{air}$, primarily determine the gain magnitude. Moreover, $Z_{L}$ mainly determines the passband characteristics of the NF-IBCC system gain. Moreover, a simplified circuit model containing only core parameters is proposed to accurately capture the gain characteristics of the NF-IBCC system. This work lays out a theoretical foundation for developing efficient and reliable NF-IBCC systems that can support IoB for early disease detection and prevention in healthcare applications.

The key contributions of this paper are as follows:

(1) We reveal the physical mechanisms of the differences in the magnitude and passband characteristics of NF-IBCC channel characteristics in existing research work through the core parameters that determine the gain of the NF-IBCC system. Specifically, $C_{H}$, and particularly $C_{air}$, primarily determine the gain magnitude. Moreover, $Z_{L}$ mainly determines the passband characteristics of the NF-IBCC system gain.

(2) The core parameters that determine the gain of the NF-IBCC system are extracted through the combination of transfer functions, finite element simulations, and physical experiments. The core parameters include the inter-body coupling capacitance ($C_{H}$), the load impedance ($Z_{L}$), and the capacitance ($C_{air}$), coupled by two floating transceiver grounds.

(3) We propose a simplified equivalent circuit model that only contains core parameters, which can accurately capture the gain characteristics of the NF-IBCC system and help to concisely describe the channel characteristics of the system.

The rest of the paper is organized as follows: Section 2 describes the fundamental theory of NF-IBCC and proposes an equivalent circuit model. The core parameters of NF-IBCC are derived and retrieved based on the equivalent circuit analysis. In Section 3, finite element analysis and physical experiments are conducted to verify the theoretical analysis. Section 4 provides a further discussion and summary. Section 5 concludes our paper.

## 2. Retrieval and Analysis of Core Parameters Based on the Transfer Function Method

The effect of the human body on electromagnetic waves is a complex phenomenon that occurs when the human body is in close proximity to a source. In addition to simple reflection, diffraction, and near-field dielectric and ohmic loss, there are other mechanisms that come into play, such as surface waves or lossy waveguide effects [21].

**Figure 2:**
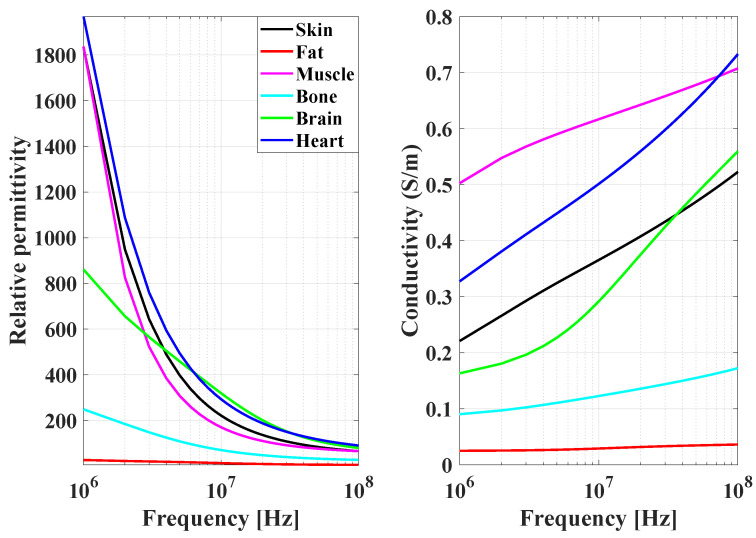
Relative permittivity and conductivity of various tissues in the human body, including skin, fat, muscle, bone, brain, and heart.

Figure 2 displays the dielectric properties of various human body tissues, including relative dielectric constant and conductivity, from 1 to 100 MHz, calculated using the Cole–Cole formula [22]. Notably, the relative permittivity of these tissues is exceptionally high, particularly below 10 MHz, indicating their strong ability to receive an electric field in this frequency range. When an electrode source is in contact with the body, the output electric field can generate surface waves that attach to the body’s surface [23]. As a result, the body and the source form a larger radiation source whose electromagnetic fields in the radiating far-field region can be ignored at frequencies of 1 to 10 MHz, as the body and electrodes are much smaller than the wavelength. Therefore, the electromagnetic fields emitted by the system, composed of electrodes and the human body, are limited to the near-field region [16].

**Figure 3:**
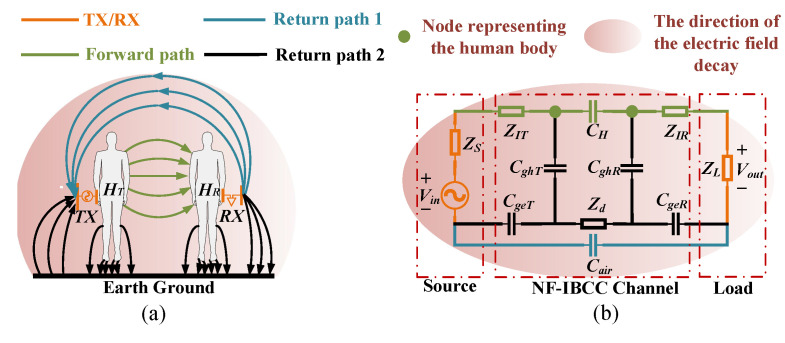
The NF-IBCC system. (**a**) Schematic diagram of signal transmission. (**b**) Equivalent circuit model.

According to our previous research results [13], a sphere-like area surrounding the TX in the presence of the bodies is defined as the near-field region. A strong coupling between two bodies occurs when the body of RX approaches the near-field region. This means that the electrodes (TX and RX) and the bodies constitute the NF-IBCC system collectively, as shown in Figure 3a. On the other hand, the induced electric fields created by the magnetic fields in this coupling system are ignored since no closed coupling loops exist at the TX or RX electrodes. Therefore, the electric field coupling predominates in this system. This inter-body coupling can be characterized as lumped capacitive coupling.

The NF-IBCC system is equivalent to a circuit model composed of lumped parameters; its accuracy was verified in our previous work, as shown in Figure 3b [13]. It consists of the forward coupling path, return-coupling path 1, and return-coupling path 2. The electric field coupling between the two bodies is defined as the forward coupling path and is represented by the lumped parameter $C_{H}$. The electric field coupling between transceiver grounds is defined as the return-coupling path 1 and is represented by the lumped parameter $C_{air}$. The electric field coupling between the transceiver ground and the earth ground is represented by the lumped parameters $C_{geT}$ and $C_{geR}$. The electric field coupling between the foot of the bodies and the earth ground is indicated by the lumped parameters $C_{ghT}$ and $C_{ghR}$. The lumped parameter $Z_{d}$ represents the loss impedance of the earth ground. $C_{geT}$, $C_{ghT}$, $C_{ghR}$, $C_{geR}$, and $Z_{d}$ represent the return-coupling path 2. In addition, $Z_{IT}$ and $Z_{IR}$ represent the skin–electrode contact impedances. The source is composed of $V_{in}$ and $Z_{S}$. $Z_{S}$ is the impedance of the source. $Z_{L}$ represents the load impedance at the receiving end.

The gain between any two devices is influenced by various factors, including the channel characteristics and the output and input impedances of the transmitter and receiver [24]. For the NF-IBCC system, the channel characteristics are determined by the forward path, return path 1, and return path 2. Therefore, this section analyzes the effects of the forward path, return path 1, return path 2, and the output and input impedances of the transmitter and the receiver on the gain of the NF-IBCC system from the perspective of circuit theory.

In addition, when there is no obstacle between the transmitter and receiver, the equivalent circuit model of the NF-IBCC channel presents a symmetrical structure because of $Z_{IT}\approx Z_{IR}$, $C_{ghT}\approx C_{ghR}$, and $C_{geT}\approx C_{geR}$. For a regular off-the-shelf RF device, the output and input impedance of the transmitter and the receiver would be 50 Ω; this is our default analysis and measurement mode. Therefore, the input impedance $Z_{in}$ of this equivalent circuit is approximately equal to the output impedance $Z_{out}$. In this circuit model, the voltage gain $Gain_{voltage}$ is approximately equal to the power gain $Gain_{power}$ caused by $Gain_{power}=10log10\left(\frac{V_{out}^{2}}{V_{in}^{2}}\cdot \frac{Z_{in}}{Z_{out}}\right)\approx 20log10\left(\frac{V_{out}}{V_{in}}\right)=Gain_{voltage}$. Hence, the voltage transfer function is used in the following circuit analysis.

### 2.1. The Forward Path

**Figure 4:**
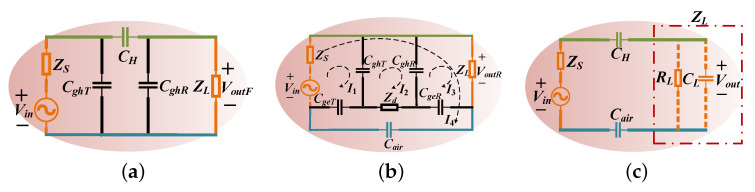
(**a**) The equivalent circuit model for the forward path. (**b**) The equivalent circuit model for the return path. (**c**) The simplified equivalent circuit model for the full channel with loads of $C_{L}$ or $R_{L}$.

In order to research the effect of the forward path on the gain of the NF-IBCC system separately, we directly connect the grounds of the transceiver to obtain the circuit model with only the forward path, as shown in Figure 4a. It only reflects the effect of the forward path impedance on the NF-IBCC system; the return path effect is eliminated. The skin–electrode contact impedance is neglected due to the lower impedance value compared to the impedance of $\left|\frac{1}{sC_{H}}\right|$ within the ≤10 MHz frequency regime [25].

According to the circuit principle, the transfer function of the forward path is obtained, as shown in Equation (1).

(1) $$\frac{V_{outF}}{V_{in}}=\frac{\frac{1}{sC_{ghT}}//\left(\frac{1}{sC_{H}}+\frac{1}{sC_{ghR}}//Z_{L}\right)}{Z_{S}+\frac{1}{sC_{ghT}}//\left(\frac{1}{sC_{H}}+\frac{1}{sC_{ghR}}//Z_{L}\right)}\cdot \frac{\frac{1}{sC_{ghR}}//Z_{L}}{\frac{1}{sC_{H}}+\frac{1}{sC_{ghR}}//Z_{L}}$$

Due to the minimal variation in distance and the area between the foot and the earth ground, the coupling capacitance between the human body and the earth ground at the transmitting end is approximately equal to that of the receiving end. Therefore, $C_{ghT}\approx C_{ghR}=C_{gh}$ can be used. The gain of the forward path can be derived as shown in Equation (2).

(2) $$Gain_{F}=20log10\left(\frac{Z_{L}{\frac{1}{sC_{gh}}}^{2}}{\left(Z_{L}+\frac{1}{sC_{gh}}\right)\left[Z_{S}+\frac{\frac{1}{sC_{gh}}\left(\frac{1}{sC_{H}}+\frac{Z_{L}\frac{1}{sC_{gh}}}{Z_{L}+\frac{1}{sC_{gh}}}\right)}{\frac{1}{sC_{H}}+\frac{1}{sC_{gh}}+\frac{Z_{L}\frac{1}{sC_{gh}}}{Z_{L}+\frac{1}{sC_{gh}}}}\right]\left(\frac{1}{sC_{H}}+\frac{1}{sC_{gh}}+\frac{Z_{L}\frac{1}{sC_{gh}}}{Z_{L}+\frac{1}{sC_{gh}}}\right)}\right)$$

Since the value of $C_{H}$, $C_{gh}$ is ∼10 pF [13], the value of $\left|\frac{1}{sC_{H}}\right|$, $\left|\frac{1}{sC_{ghR}}\right|$ is greater than or equal to 1 kΩ within the ≤10 MHz frequency regime. The output and input impedance of the transmitter and the receiver is equal to 50 Ω. Consequently, the power gain can be derived using Equation (3).

(3) $$\begin{array}{cc} Z_{S} & =Z_{L}=50\Omega \ll \left|\frac{1}{sC_{H}}\right|,\left|\frac{1}{sC_{gh}}\right|;\\ Gain_{F} & \approx 20log10\left(\frac{Z_{L}}{\frac{1}{sC_{H}}}\right)=20log10\left(j2\pi fZ_{L}C_{H}\right) \end{array}$$

where the value of $C_{H}$ can be obtained by the simulation based on the Ansys Maxwell 3D platform. The simulation setup is as described in Section 3.1. By combining the simulated curve and the results from previous works [26,27], we can derive a fitting equation that describes the relationship between $C_{H}$ and the relative distance *d* (m), and the surface area of the body BSA, as shown in Equation (4), where $\varepsilon_{0}$ is the vacuum permittivity, and $\varepsilon_{r}$ is the relative permittivity of the substance between the human bodies. The value of BSA is determined by the smaller surface area in the two human bodies.

(4) $$C_{H}=\left[1+5.257{\left(\frac{d}{h}\right)}^{1.083}\right]\times \frac{\varepsilon_{0}\varepsilon_{r}BSA}{2d}$$

The surface area of the body BSA can be obtained from Equation (5) [28] by knowing the height *h* and weight *W*.

(5) $$BSA=71.3989\times h^{0.7437}\times W^{0.4040}$$

where *h* is the height of the human body (cm), *W* is the weight of the human body (kg), and the unit of the surface area BAS of the human body is in square centimeters (cm^2^).

According to Equations (3)–(5), $C_{H}$ is the core parameter of the forward path. This indicates that the gain of the forward path primarily depends on the coupling capacitance $C_{H}$. The power leaked to the earth ground through the coupling capacitance $C_{gh}$ is considered negligible [29].

On the other hand, when there is no barrier between the two bodies, the impact of the core parameter $C_{H}$ on the gain of the forward path is specifically manifested in the fact that the gain is affected by the distance (*d*) and the surface area of the body (BSA). As the distance between the two bodies increases, meaning that the receiving end is further from the near-field range of the electrodes and the human body forming the radiator, the gain decreases, which is obvious. Moreover, individuals with higher heights and heavier weights have larger surface areas, resulting in greater values of the coupling capacitance $C_{H}$, and ultimately increasing the gain. Therefore, the participation of individuals with higher heights and heavier weights in the NF-IBCC system can be more advantageous for information transmission. Furthermore, when the load impedance $Z_{L}$ of the receiving end is 50 Ω, the gain increases with the *f*, indicating that the channel of the forward path exhibits high-pass characteristics.

### 2.2. The Return Path

This subsection discusses the gain of the return path. In order to study the gain of the return path and eliminate the influence of the forward path, the equivalent circuit model of the return path can be obtained by connecting the signal electrodes of the transmitting end and the receiving end through wires, as shown in Figure 4b.

Given that the source impedance $Z_{S}$ = 50 Ω and the input impedance of the NF-IBCC channel are capacitive, it is worth noting that the value of $Z_{S}$ is significantly smaller than the input impedance in the frequency range of 1–10 MHz. Therefore, we can omit $Z_{S}$ in this case. According to the circuit model, the voltage in each mesh is calculated by using KCL to achieve four linear equations in a matrix form, as shown in Equation (6).

(6) $$\left[\begin{array}{cccc} \frac{1}{sC_{ghT}}+\frac{1}{sC_{geT}} & -\frac{1}{sC_{ghT}} & 0 & 0\\ -\frac{1}{sC_{ghT}} & \frac{1}{sC_{ghT}}+\frac{1}{sC_{ghR}}+Z_{d} & -\frac{1}{sC_{ghR}} & 0\\ 0 & -\frac{1}{sC_{ghR}} & \frac{1}{sC_{ghR}}+\frac{1}{sC_{geR}}+Z_{L} & Z_{L}\\ 0 & 0 & Z_{L} & Z_{L}+\frac{1}{sC_{air}} \end{array}\right]\left[\begin{array}{c} I_{1}\\ I_{2}\\ I_{3}\\ I_{4} \end{array}\right]=\left[\begin{array}{c} V_{in}\\ 0\\ 0\\ V_{in} \end{array}\right]$$

Assuming that the relative area and distance between the earth ground and the ground of the transceiver are equal, $C_{geT}=C_{geR}=C_{ge}$ can be used. In addition, as $C_{air}$ and $C_{ge}$ are both less than 10 pF [13], the values of $\left|\frac{1}{sC_{air}}\right|$ and $\left|\frac{1}{sC_{ge}}\right|$ are greater than 10 kΩ. However, $Z_{L}$ = 50 Ω, the value of $Z_{d}$ is ∼10 Ω [13]. As a consequence, Equation (6) can be further derived as Equation (7).

(7) $$\begin{array}{cc} Z_{L} & =50\Omega ,Z_{d}\ll \left|\frac{1}{sC_{air}}\right|,\left|\frac{1}{sC_{ge}}\right|,\left|\frac{1}{sC_{gh}}\right|;\\ \; & \left[\begin{array}{cccc} \frac{1}{sC_{gh}}+\frac{1}{sC_{ge}} & -\frac{1}{sC_{gh}} & 0 & 0\\ -\frac{1}{sC_{gh}} & 2\frac{1}{sC_{gh}} & -\frac{1}{sC_{gh}} & 0\\ 0 & -\frac{1}{sC_{gh}} & \frac{1}{sC_{gh}}+\frac{1}{sC_{ge}} & Z_{L}\\ 0 & 0 & Z_{L} & \frac{1}{sC_{air}} \end{array}\right]\left[\begin{array}{c} I_{1}\\ I_{2}\\ I_{3}\\ I_{4} \end{array}\right]=\left[\begin{array}{c} V_{in}\\ 0\\ 0\\ V_{in} \end{array}\right] \end{array}$$

The transfer function of the return path is shown in Equation (8).

(8) $$\frac{V_{outR}}{V_{in}}=\frac{\left(I_{3}+I_{4}\right)Z_{L}}{V_{in}}$$

Equation (8) can be approximated as Equation (9).

(9) $$\begin{array}{cc} \frac{V_{outR}}{V_{in}} & \approx \frac{Z_{L}\left(2{\frac{1}{sC_{ge}}}^{2}+2\frac{1}{sC_{ge}}\frac{1}{sC_{gh}}+\frac{1}{sC_{air}}\frac{1}{sC_{gh}}\right)}{\frac{1}{sC_{air}}\left(2{\frac{1}{sC_{ge}}}^{2}+2\frac{1}{sC_{ge}}\frac{1}{sC_{gh}}\right)}\\ \; & =\frac{Z_{L}}{\frac{1}{sC_{air}}}+\frac{Z_{L}\frac{1}{sC_{gh}}}{2{\frac{1}{sC_{ge}}}^{2}+2\frac{1}{sC_{ge}}\frac{1}{sC_{gh}}} \end{array}$$

The gain of the return path can be derived as Equation (10).

(10) $$Gain_{R}=20log10\left[j\omega Z_{L}\left(\frac{C_{ge}^{2}}{2\left(C_{ge}+C_{gh}\right)}+C_{air}\right)\right]$$

From Equation (10), the following conclusion can be obtained:

(11) $$\frac{C_{ge}^{2}}{2\left(C_{ge}+C_{gh}\right)}\ll C_{air}.$$

Therefore, the power gain of the return path can be approximated as follows:

(12) $$Gain_{R}\approx 20log10\left(j2\pi fZ_{L}C_{air}\right).$$

Similarly, the value of $C_{air}$ can be obtained by simulations based on the Ansys Maxwell 3D platform. The simulation setup is as described in Section 3.1. Furthermore, the fitting equation describing the relationship between $C_{air}$, the relative distance *d* (m), and the area $S_{air}$ (m^2^) is also obtained, as shown in Equation (13), where $\varepsilon_{0}$ is the vacuum permittivity, $\varepsilon_{r}$ is the relative permittivity of the substance between transceiver grounds, and *l* (m) represents the longer side length of the ground of the transceiver.

(13) $$C_{air}=\left[1+0.8868{\left(\frac{d}{l}\right)}^{0.9362}\right]\times \frac{\varepsilon_{0}\varepsilon_{r}S_{air}}{d}$$

From Equation (12), it can be found that the core parameter of the return path is $C_{air}$, which determines the gain of the return path. At the same time, it can be seen from Equation (11) that the path loss caused by the leakage of the electromagnetic field distributed on the human body to the earth ground through $C_{ghT}$ and $C_{ghR}$ can be ignored. Consequently, the influence of the return path 2 on the return path can be ignored.

In addition, it can be seen from Equation (13) that when there is no obstacle between the ground of the transmitting end and the receiving end, the influence of the core parameter $C_{air}$ on the gain of the return path can be expressed as the gain being affected by the distance (*d*) and the relative area ($S_{air}$). Specifically, as *d* increases, the gain decreases. However, when the NF-IBCC system is implemented with a larger ground area for both the transmitter and receiver, it results in an increase in gain. This increased gain can be effectively utilized to enhance the signal-to-noise ratio of the NF-IBCC system. It is important to note that the transmitter and receiver in the NF-IBCC system are wearable devices and have size limitations. Therefore, it is not feasible to have an infinite ground area for the transmitter and receiver.

Furthermore, it is worth emphasizing that when measuring the channel characteristics of the NF-IBCC system, it is crucial to employ a ground-separated instrument. Moreover, the ground area of the measuring instrument should closely match the ground area of the transceiver in the real-world application. By ensuring that the ground areas are well-matched, it is possible to obtain channel characteristics that are relatively accurate and representative of the system’s performance. On the contrary, if a common-ground instrument is used, such as a vector network analyzer, it can be considered that the coupling capacitance $C_{air}$ is short-circuited, which will inevitably lead to more optimistic channel characteristics.

Similarly, Equation (12) shows that when the load impedance at the receiving end is 50 Ω, the gain increases with the increase in frequency *f*, which means that the channel of the return path has high-pass characteristics.

### 2.3. The Impedance of Source and Load

In a practical application scenario, one needs to consider the source and load impedance present at the transmitter and the receiver, respectively. In the following subsection, we will go through a few special cases of source and load impedances. Due to the selection of source and load impedances in special cases, the input impedance $Z_{in}$ and the output impedance $Z_{out}$ are not equal, which further causes the voltage gain $Gain_{voltage}$ to be not equal to the power gain $Gain_{power}$. However, in this subsection, we will use voltage gain to examine the impacts of the source and load impedances on the gain characteristics. The corresponding simulations and experiments in the following part also use the method of measuring the voltage gain to verify the theoretical analysis.

As mentioned in the previous subsection, the path loss caused by the return path 2 can be disregarded, implying that the gain of the return path largely hinges on the coupling capacitance $C_{air}$. In Section 2.1, it was indicated that the coupling capacitance $C_{H}$ is the primary determinant of the gain of the forward path. Consequently, the NF-IBCC channel can be further simplified as a series circuit, composed of the coupling capacitance $C_{H}$ and $C_{air}$, as shown in the lumped circuit model depicted in Figure 4c. If the source impedance is significantly smaller than the input impedance, the input voltage of the channel can be as large as possible, facilitating the voltage signal transmission. Generally, measuring instruments have an output impedance of 50 Ω. However, the input impedance in this circuit model, $Z_{in}=\frac{1}{sC_{H}}+\frac{1}{sC_{air}}+Z_{L}$, already fulfills the condition of $Z_{in}\gg Z_{S}$. Thus, we will not elaborate much on the source impedance in this discussion.

The special load impedance cases are highlighted in this subsection. From the circuit model shown in Figure 4c, the voltage transfer function can be obtained using Equation (14).

(14) $$\frac{V_{out}}{V_{in}}=\frac{Z_{L}}{Z_{S}+\frac{1}{sC_{H}}+Z_{L}+\frac{1}{sC_{air}}}$$

When the load impedance is resistance and has a value of 50 Ω, the gain can be derived as Equation (15).

(15) $$\begin{array}{cc} Z_{L} & =R_{L}=50\;\Omega ,Z_{S}=50\;\Omega \ll \left|\frac{1}{sC_{air}}\right|,\left|\frac{1}{sC_{H}}\right|;\\ Gain & =20log10\left(\frac{Z_{L}}{\frac{1}{sC_{H}}+\frac{1}{sC_{air}}}\right)=20log10\left(\frac{j2\pi fZ_{L}C_{H}C_{air}}{C_{H}+C_{air}}\right) \end{array}$$

Equation (15) reveals that when the load impedance $Z_{L}$ = $R_{L}$ = 50 Ω, the gain of the NF-IBCC system increases with an increase in frequency (*f*), indicating a high-pass characteristic of the channel. Moreover, Equations (3) and (12) demonstrates that both the forward path and return path of the NF-IBCC system exhibit high-pass characteristics, and the loop formed by these paths also has a noticeable high-pass characteristic. Hence, the results provided by Equation (15) align with Equations (3) and (12).

Furthermore, the gain of the NF-IBCC system depends on the series-equivalent capacitance of the coupling capacitances $C_{H}$ and $C_{air}$. The influence of the coupling capacitance with the smaller value has a more significant effect on the gain. Comparing Equations (4) and (13), it can be seen that the value of $C_{H}$ is much larger than $C_{air}$. This indicates that the gain of the NF-IBCC system is more dependent on the core parameter $C_{air}$, further supporting the perspective that the channel characteristics of NF-IBCC cannot be accurately measured using common ground instruments.

When the load impedance is a capacitance, the gain can be derived using Equation (16).

(16) $$\begin{array}{cc} Z_{L} & =\frac{1}{sC_{L}},Z_{S}=50\;\Omega ;\\ Gain & =20log10\left(\frac{C_{H}C_{air}}{C_{L}C_{air}+C_{H}C_{L}+C_{H}C_{air}}\right) \end{array}$$

From Equation (16), it is apparent that the gain of the NF-IBCC system is independent of the operating frequency (*f*) but solely determined by the values of the core parameters, $C_{H}$ and $C_{air}$, and the load capacitance, $C_{L}$. This implies that when the load impedance is given by $Z_{L}=\frac{1}{sC_{L}}$, the channel of NF-IBCC exhibits a flat-band characteristic. Moreover, apart from the dependence on the series-equivalent capacitance of the coupling capacitances, $C_{H}$ and $C_{air}$, the gain is also influenced by the load capacitance, $C_{L}$. Specifically, a smaller value of $C_{L}$ results in a higher gain. This observation provides an avenue for enhancing the system gain of NF-IBCC.

## 3. Simulation and Experimental Results

In this section, we validate the theoretical results mentioned above using finite element simulations and physical experiments.

### 3.1. Simulation Setup

The simulations were carried out using Ansys HFSS and Maxwell, which are finite elements analysis (FEA) tools. HFSS is an EM solver used for high-frequency applications whereas Maxwell is used in our simulations as an electrostatic solver.

**Figure 5:**
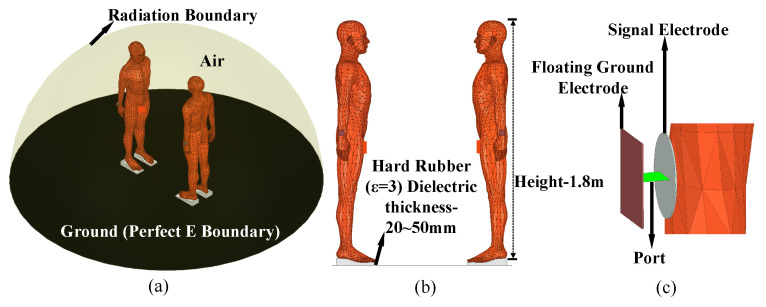
The finite element simulation setup for the NF-IBCC system: (**a**) the overall view of the finite element model. (**b**) A human body model with a height of 1.8 m has a rubber layer with a thickness ranging from 20 to 50 mm between its feet and the earth ground. (**c**) The electrode structure is mainly composed of a signal electrode, ground electrode, and port.

For our simulations, we utilized a single-layer human body model available in the HFSS accessory library, as shown in Figure 5b. The height of the model was 1.8 m. The relative permittivity and conductivity used in the model were adapted from Gabriel et al. [22]. The dielectric properties were set to be the frequency-dependent average of human tissue properties throughout the body, weighted by their relative volumes. The validity of using the average electric properties of the tissue in our simulations was confirmed by previous literature [21]. The simulation results obtained using these properties were found to be sufficiently similar to those obtained using a multi-layered model. In order to replicate an infinite earth ground plane, a plane with a perfect E boundary was placed beneath the human body. A rubber layer with a thickness of 20–50 mm was located between the foot and the earth ground plane. Two identical human bodies were positioned facing each other. All models were placed in a hemispherical air medium with a radius of 1.5 m, as shown in Figure 5a. The surface of the hemisphere was set as a radiation boundary condition.

**Figure 6:**
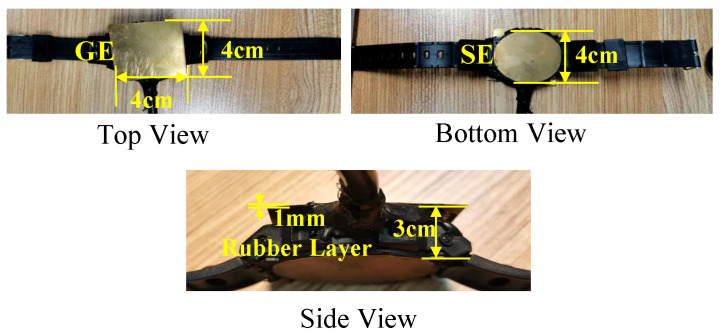
Electrodes used in the physics experiments.

The excitation model is composed of a signal electrode, ground electrode, and port, as shown in Figure 5c. The signal electrode is represented by a circular copper sheet with a radius of 2 cm and a thickness of 1 mm. This is consistent with the material and size of the signal electrode in the experimental setup shown in Figure 6. The ground electrode is represented by a square copper sheet with a thickness of 1 mm, and its specific size can be set according to the size of the ground of the transceiver in the experimental setup. The signal electrode is in contact with the skin of the wrist, while the ground electrode is suspended in the air. In the simulation, the lumped port and voltage port are utilized to calculate the power gain and voltage gain, respectively. When using the lumped port, both the transmitter and receiver ports are defined as square faces of the PEC and terminated with a 50 Ω load. When using the voltage port, the ports at the transmitter and receiver are defined as lumped RLC boundary conditions. The transmit port is given an AC voltage difference with an amplitude of 1 V, while the potential difference at the receive port is obtained by integrating the electric field along a straight line between the signal electrode and the ground electrode. The simulations were performed at a frequency range of 1 to 10 MHz.

On the other hand, the inter-body coupling capacitance $C_{H}$ and the coupling capacitance $C_{air}$ were obtained through simulations in ANSYS Maxwell, which is a finite element method (FEM)-based solver for static Maxwell equations. The simulations were conducted in Maxwell’s electrostatic mode, using the same two human body models to calculate the capacitance matrix between the two bodies by treating them as individual conducting objects. Specifically, two face-to-face human bodies were placed in the air box as described in Figure 5a. The human body is regarded as an electrode plate; the voltage of the human body at the transmitting end was set to 1 V, while the voltage of the human body at the receiving end was 0 V. The capacitance value between the two human bodies can be obtained through simulation. Similarly, a two-plate metal model was utilized to simulate and calculate the value of $C_{air}$.

### 3.2. Experimental Setup

All experiments were conducted inside the teaching building. We employed three different types of gain measurement setups: (1) A vector network analyzer (the KEYSIGHT E5071C setup has a frequency range of 100 kHz to 8.5 GHz, and the port impedance is 50 Ω) with the transmit and receive ends common-grounded to short-circuit the return path of the NF-IBCC, thereby eliminating any return path effect; (2) a handheld signal generator (the Ceyear1431A setup has a frequency range of 250 kHz to 4 GHz, with an output power range from −120 to +5 dBm, and an output impedance of 50 Ω; its physical dimensions are 33 cm × 23 cm × 8.5 cm) and handheld spectrum analyzer (the Ceyear4957B setup has a frequency range of 9 kHz to 6.5 GHz, an average noise level of ≤−135 dBm, and an input impedance of 50 Ω; its physical dimensions are 31.5 cm × 22 cm × 10.2 cm), which completely isolate the ground of the transmitter and receiver to replicate the actual NF-IBCC scenario; (3) a handheld signal generator and oscilloscopes (KEYSIGHT infiniiVision MSOX4154A, 1.5 GHz, 5 GSa/s) that provide measurements of the voltage gain with high-impedance capacitive terminations. The measurement setup of the system is discussed in the next subsections.

The transmitting end is set to sweep frequencies in the range of 1 to 10 MHz, with an output power of 0 dBm, which is well below the safety limit set by ICNIRP [15]. The electrode (as shown in Figure 6) consists of a signal electrode (SE), which has a thickness of 1 mm and a radius of 2 cm, a ground electrode (GE), which has a thickness of 1 mm and an area of 4 cm × 4 cm, and a rubber layer (3 cm thickness) located between the SE and GE to isolate them and prevent short circuits. The electrode is fixed on the wrist using a watchband, with the SE in contact with the skin and the GE suspended in the air.

### 3.3. The Forward Path

In order to study the gain of the forward path, the return path needs to be shorted to eliminate the effect coming from the return path.

**Figure 7:**
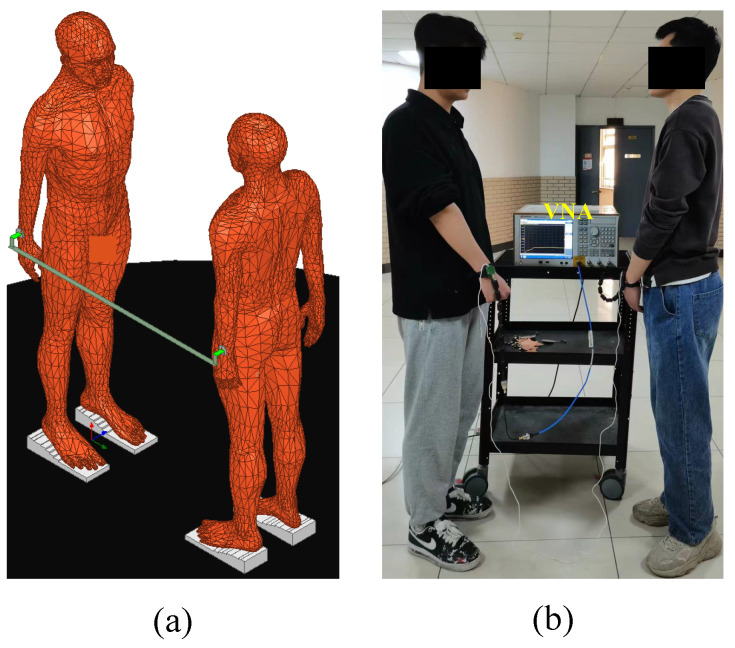
(**a**) The finite element model of the forward path in the NF-IBCC system. (**b**) The physical experimental setup of the forward path in the NF-IBCC system.

The finite element model, as illustrated in Figure 7a, features a PEC plate (the impedance of the PEC plate is approximately zero) connecting the ground of the transmitting end and the ground of the receiving end to achieve a short-circuit effect. The signal electrode (a PEC disc with a thickness of 1 mm and a radius of 2 cm) is kept in contact with the skin of the wrist. The signal is then transmitted from the transmitting end to the receiving end through the inter-body coupling capacitance $C_{H}$, detected by the receiving end signal electrode, and passed through the PEC plate to return to the transmitter, forming a loop that is consistent with the circuit model, as shown in Figure 4a. In addition, both the transmitter and receiver ports in Figure 7a are set as lumped ports with a load impedance of 50 Ω. The input power of the transmitter port is set to 0 dBm (1 mW). The power gain between the transmitting end and the receiving end, i.e., the S21 parameter, can be obtained through simulation.

In Figure 7b, a VNA is employed to measure the gain. Although the transmitter ground and the receiver ground of the VNA are shorted, wires are connected between the two ground electrodes to further eliminate any impact of the return path. The two bodies have similar physical characteristics in terms of height and weight, both measuring approximately 1.80 m and weighing around 80 kg.

**Figure 8:**
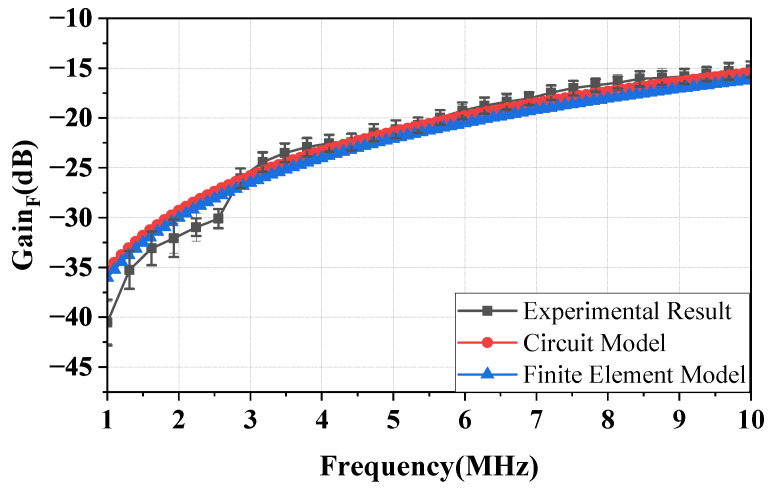
The forward path gain of the NF-IBCC system at a distance of 0.3 m, including the circuit model simulation results, finite element simulation results, and experimental results.

Figure 8 illustrates the gain of the forward path of the NF-IBCC in the frequency range of 1–10 MHz when the distance is *d* = 0.3 m. Comparative experiments, the EM simulations of an identical setup in HFSS, and Equation (3) yield consistent results, indicating good agreement among the three cases. The observed consistency in the results can be attributed to keeping the core parameter (specifically, the forward path gain is determined by the distance parameter (d) and the surface area, which depend on the height (h) and weight (W)) to be as consistent as possible across the three cases. This confirms the previous analysis, which showed that $C_{H}$ is the core parameter of the forward path, and the gain of the forward path is mainly determined by $C_{H}$. In addition, when the source and load impedances are both 50 Ω, the forward path channel has the characteristics of a high-pass filter. Specifically, the gain increases gradually from −35 to −15 dB at a distance of *d* = 0.3 m. In addition, gains are measured for three consecutive days, each morning, noon, and night. It is found that the variation of gain within the 3 MHz range is within 2.5 dB, and the variation of gain within the 3–10 MHz range is within 1.5 dB. This may be due to the weak resolution of the VNA at low frequencies [30]. This also illustrates the reproducibility of the experimental results.

**Figure 9:**
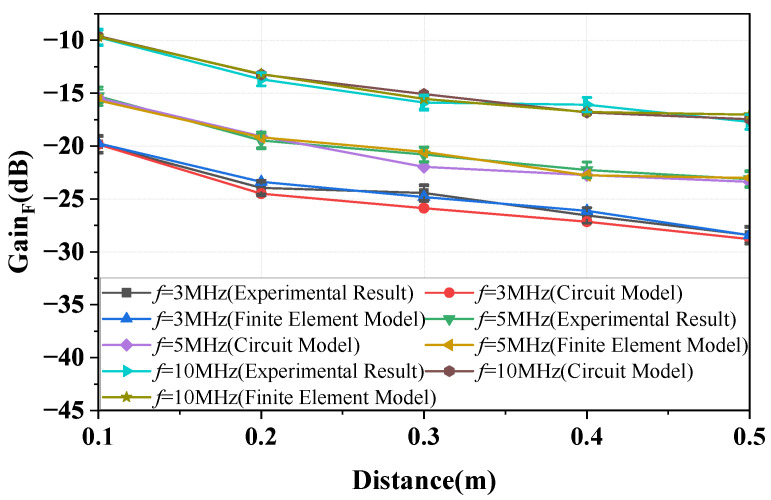
The change curve of the forward path gain of the NF-IBCC system with distance obtained through the circuit model analysis, finite element simulation, and physical experiment measurements.

According to Equation (4), $C_{H}$ is primarily determined by the distance *d* and the relative area between the bodies. Figure 9 shows that at frequencies of 3, 5, and 10 MHz, the gain of the forward path decreases as the distance *d* increases. The figure shows that the physical experiment results and finite element simulation results are consistent with the predictions of Equations (3) and (4). In addition, the results show that the measured gain (over multiple days) exhibits a variation of within 1.5 dB.

**Figure 10:**
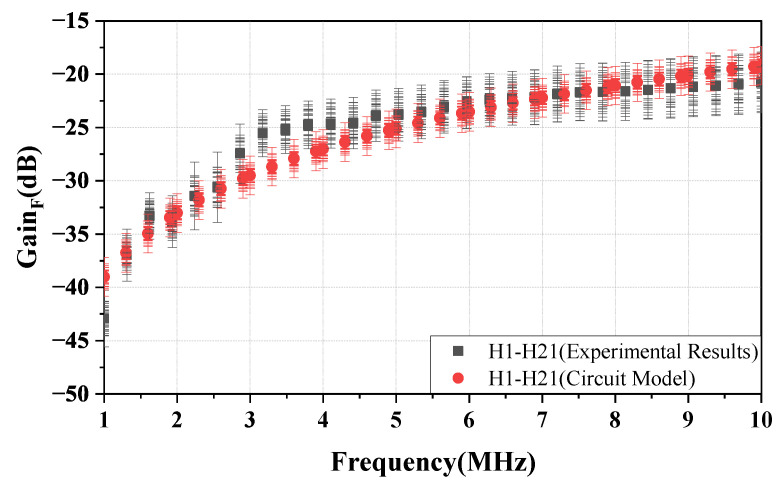
At a distance of 0.4 m, a total of 21 volunteers (13 males and 8 females) were paired and used to obtain the dynamic variation range of the forward path gain through physical experimental measurements and circuit model analysis.

On the other hand, the height and weight of the body determine the relative area, which in turn affects the gain of the forward path. To confirm this relationship, we recruited 21 subjects (13 males and 8 females) with weights and heights ranging from 50 to 92 kg and 1.57 to 1.90 m, respectively. The subjects were paired up for measurements. Figure 10 shows the gain of the forward path at a distance of *d* = 0.4 m. Based on the experimental results, the average dynamic range of the gain caused by variations in height and weight is 4.43 dB. In contrast, the average dynamic range of gain predicted by Equations (3)–(5) is 2.88 dB. The difference between the two values is 1.55 dB, indicating that Equations (3)–(5) can partially account for the gain variations caused by height and weight.

### 3.4. The Return Path

**Figure 11:**
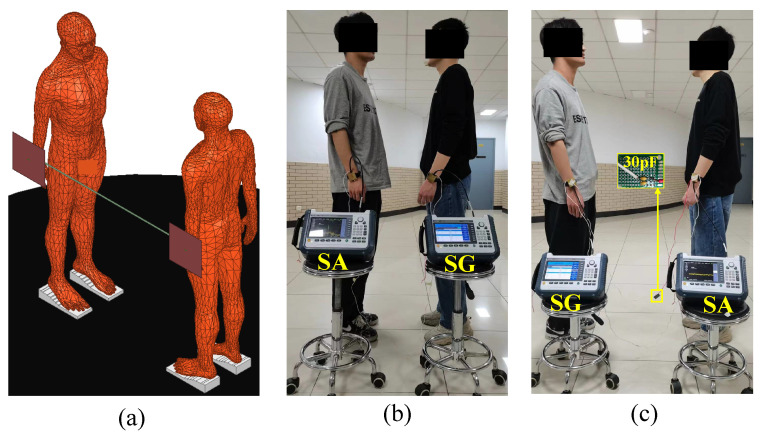
The setup for the return path in the NF-IBCC system: (**a**) finite element simulation setup, (**b**) physical experiment setup, (**c**) the physical experimental setup after replacing the return path with a 30pF capacitor.

To investigate the gain of the return path, the forward path can be short-circuited by directly connecting the signal electrodes of the transmitter and receiver with wires to eliminate any impact from the forward path. Figure 11a presents the finite element model of the return path, where the signal electrodes at the transmitting and receiving ends are connected by a PEC plate to simulate a short-circuit of the forward path. Moreover, the ground planes at the transmitting and receiving ends are modeled as PEC plates with dimensions of 33 cm × 23 cm × 1 mm and 31.5 cm × 22 cm × 1 mm, respectively, which match the physical dimensions of the handheld signal generator and the handheld spectrum analyzer. Furthermore, in Figure 11a, both the transmitter and receiver ports are configured as lumped ports with a 50 Ω load. The transmitter port is set with an input power of 0 dBm (1 mW). The power gain from the transmitting end to the receiving end can be determined by simulating the S21 parameter in HFSS.

Figure 11b shows the experimental setup for the return path, which uses a handheld signal generator and a handheld spectrum analyzer as the transmitter and receiver, respectively. The use of these devices helps isolate the ground. Furthermore, wires are employed to connect the signal electrodes at the transmitting and receiving ends, simulating a short-circuit of the forward path. In addition, as shown in Figure 11c, a 30 pF capacitor is connected between the ground of the transmitting end and the ground of the receiving end, and the signal electrodes of the transmitting and receiving ends are connected by wires. This setup is used to further verify the functionality of the return path 2 in the overall return path.

**Figure 12:**
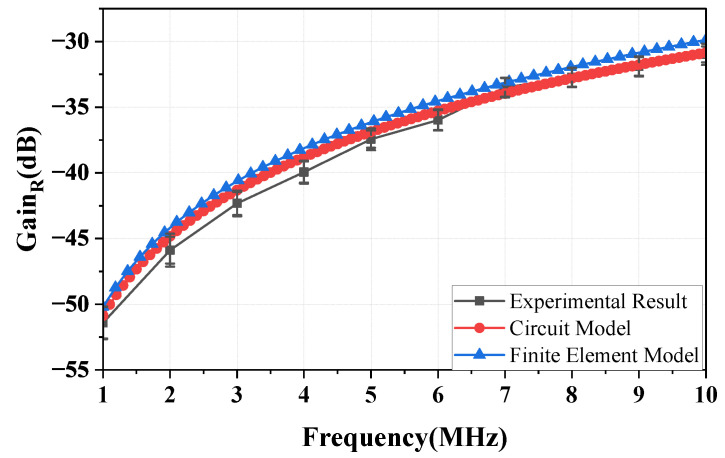
At a distance of 0.1 m, the return path gain of the NF-IBCC system is evaluated through the circuit model analysis, finite element simulation, and physical experimental measurements.

Figure 12 shows the gain of the NF-IBCC return path in the frequency range of 1–10 MHz when the distance between the bodies and the transceiver is 0.1 m. It can be observed from the figure that the experimental results, finite element simulation results, and the results predicted by Equations (12) and (13) are in good agreement. As described by Equations (12) and (13), the gain of the return path is determined by the distance (d) and the relative area (Sair) between the ground of the transceiver. The consistency of the results for the three cases above is attributed to keeping these core parameters consistent across the three cases. This indicates that the gain of the return path mainly depends on the coupling capacitor $C_{air}$, as described in Equation (12), while the gain of the return path 2 can be disregarded. Furthermore, for a distance of *d* = 0.1 m, the gain of the return path gradually increases from −51.2 to −31.25 dB, which is considerably smaller than the forward path gain at a distance of *d* = 0.3 m. This suggests that the signal strength of the return path is weak. In addition, the results show that the measured gain (over multiple days) exhibits a variation of within 2.1 dB.

To investigate the impact of the return path 2, an experiment was designed, as depicted in Figure 11c, where a 30 pF capacitor was used to replace the coupling capacitor $C_{air}$. This was done to maintain the gain of the return path 1 and to primarily study the effect of the return path 2 change on the overall gain of the return path.

**Figure 13:**
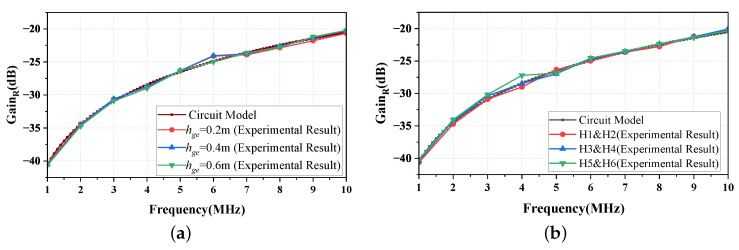
The gain of the return path in the NF-IBCC system after being replaced by a 30pF capacitor. (**a**) The gain is evaluated through the circuit model analysis and physical experimental measurements while changing one of the parameters $h_{ge}$ that determine the value of $C_{ge}$. (**b**) The gain was obtained through the circuit model analysis and physical experiment measurements while changing the parameters $S_{gh}$ and $T_{gh}$, which determine the value of $C_{gh}$.

To further study the impact of $C_{geT}$ and $C_{geR}$ on the gain in more detail, one can use the general formula $C_{geT}=C_{geR}=\frac{\varepsilon S_{ge}}{h_{ge}}$ to calculate the capacitance values, where $S_{ge}$ is the ground area of the transceiver, $h_{ge}$ is the height of the ground of the transceiver relative to the earth ground, and ε is the permittivity of the material between the ground of the transceiver and the earth ground. By changing the height $h_{ge}$ of the ground of the transceiver relative to the earth ground, the capacitance values of $C_{geT}$ and $C_{geR}$ can be altered, which in turn may affect the gain of the return path. As shown in Figure 13a, the height $h_{ge}$ of the transceiver’s ground relative to the earth ground varies from 0.2 to 0.6 m in order to measure the gain of the return path. It can be observed from the figure that even though the values of $C_{geT}$ and $C_{geR}$ were altered due to the changes in $h_{ge}$, the gain of the return path remains constant and is consistent with the one described in Equation (12) (with $C_{air}$ = 30 pF).

On the other hand, it is known that the capacitance values $C_{ghT}$ and $C_{ghR}$ can be calculated using the general formula $C_{ghT}=C_{ghR}=\frac{\varepsilon S_{gh}}{T_{gh}}$, where $S_{gh}$ is the relative area between the feet and the earth ground, $T_{gh}$ is the thickness of the material between the feet and the earth ground, and ε is the permittivity of the material. Therefore, differences in feet sizes and sole thicknesses among individuals can lead to variations in the relative area and the thickness between the feet and the earth ground; hence, this can lead to variations in $C_{ghT}$ and $C_{ghR}$, which may, in turn, affect the gain of the return path. Hence, we recruited six individuals (four men and two women) who varied in shoe size and shoe type; we conducted measurements of the return path gain. As illustrated in Figure 13b, the experimental results indicate that even though the values of $C_{ghT}$ and $C_{ghR}$ vary due to the differences in the sizes of the feet and the thicknesses of the soles among the participants, the gain of the return path remains constant and consistent with that described in Equation (12) (with $C_{air}$ = 30 pF).

In conclusion, the return path gain caused by the return path 2, composed of $C_{ghT}$, $C_{ghR}$, $C_{geT}$, $C_{geR}$, and $Z_{d}$, can be neglected, which reinforces the notion that $C_{air}$ is the fundamental parameter of the return path, and it primarily determines the gain of the return path.

**Figure 14:**
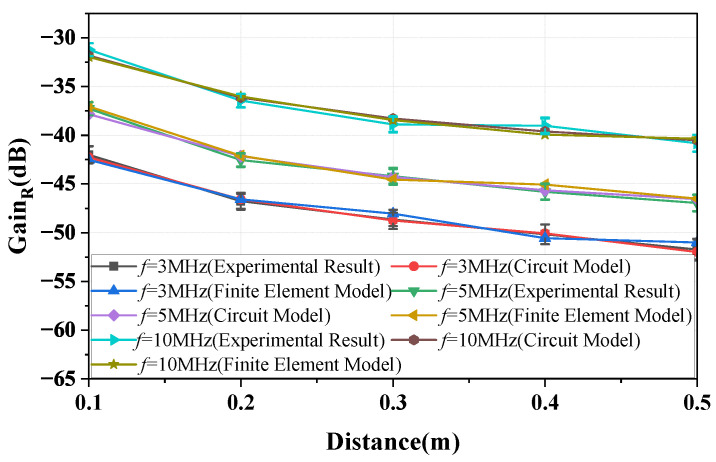
The curves showing the change in the return path gain with distance at frequencies of 3, 5, and 10 MHz are obtained through circuit analysis, finite element simulation, and physical experimental measurements, respectively.

Figure 14 depicts the variation of the gain of the return path with distance *d* at frequencies of 3, 5, and 10 MHz. As shown in the figure, the gain decreases as the distance increases, and this behavior is consistent with both the experimental and finite element simulation results, as well as the analytical results derived from Equations (12) and (13). In addition, the results show that the variation in the measured gain over multiple days is within 2.6 dB.

### 3.5. The Impedance of Source and Load

The analysis results from Section 2.3 indicate that the load impedance can significantly affect the passband characteristics of the gain of the NF-IBCC system. Specifically, a load impedance of $Z_{L}$ = 50 Ω will produce high-pass characteristics, while a load impedance of $Z_{L}=\frac{1}{j\omega C_{L}}$ will result in flat-band characteristics. To confirm these findings, finite element simulations and experimental methods are employed in this subsection.

**Figure 15:**
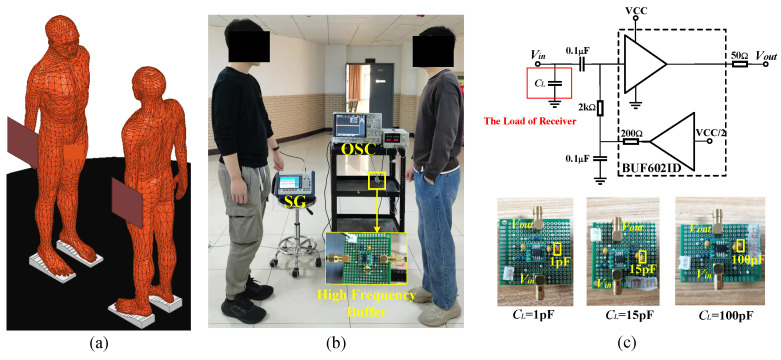
The setup used to vary the load at the receiving end of the NF-IBCC system: (**a**) The finite element setup that can change the load at the receiving end. (**b**) The experimental setup includes a signal generator and an oscilloscope and can realize the load impedance of $Z_{L}$ = 50 Ω and $Z_{L}=\frac{1}{sC_{L}}$, respectively. (**c**) The buffer setup created by Texas Instruments’ BUF602ID is used to change the load on the receiving end to $C_{L}$. The load $C_{L}$, enclosed in the red box, represents the load at the receiving end, and is configured as 1 pF, 15 pF, and 100 pF, respectively.

Figure 15a depicts a finite element model, where the ports of the transmitting and receiving ends are set to lumped RLC boundary conditions. The port impedance at the transmitting end is set to $Z_{L}$ = 50 Ω, while at the receiving end, it can be set to either $Z_{L}$ = 50 Ω or $Z_{L}=\frac{1}{sC_{L}}$. The port on the transmitter is set as a voltage port. We apply an AC voltage difference with an amplitude of 1 V ($V_{in}$ = 1 V) across the port. The voltage at the receiving end can be obtained through simulation. Specifically, this voltage ($V_{out}$) is obtained by integrating the electric field along the line between the signal electrode and the ground electrode. Finally, the voltage gain between the transmitting end and the receiving end can be determined using the equation $Gain=20log10\left(\frac{V_{out}}{V_{in}}\right)$.

The experimental setup, depicted in Figure 15b, consists of a handheld signal generator and an oscilloscope used at the transmitting and receiving ends, respectively. Both have output and input impedances of 50 Ω. To provide $Z_{L}=\frac{1}{sC_{L}}$ to the receiving end, a buffer setup is created, using the BUF602ID from Texas Instruments. As shown in Figure 15c, the load $C_{L}$, enclosed in the red box, represents the load at the receiving end, and is configured as 1 pF, 15 pF, and 100 pF, respectively, to ascertain that increasing $C_{L}$, as described in Equation (16), leads to a reduction in the gain of NF-IBCC. In addition, the buffer setup’s output load is set to a 50 Ω resistor to match the oscilloscope’s input impedance (50 Ω). Furthermore, the setup can isolate the grounds of the transceiver. The relative area between the transceiver grounds, which determines the value of $C_{air}$, is based on the smallest area of the transceiver ground, namely, the area of the ground of SG. Consequently, in the simulation model, both the transmitting and receiving end grounds are set to a PEC plate measuring 33 cm × 23 cm × 1 mm, which is consistent with the SG ground’s physical size. During the experiment, the SG and the oscilloscope are placed at the same height, following the simulation model’s settings.

**Figure 16:**
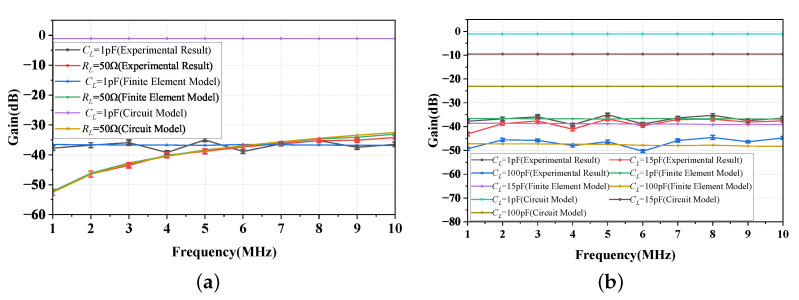
(**a**) The gain of the NF-IBCC system with loads of 50 Ω and $C_{L}$ = 1 pF is obtained through a combination of simplified circuit model analysis, finite element simulation, and physical experiment measurements. (**b**) The gain of the NF-IBCC system with loads of $C_{L}$ = 1 pF, 15 pF, and 100 pF is obtained through a combination of simplified circuit model analysis, finite element simulation, and physical experiment measurements.

In Figure 16a, the gain of the NF-IBCC system is illustrated for two load impedances, namely $Z_{L}$ = 50 Ω and $Z_{L}$ = $\frac{1}{sC_{L}}$ (with $C_{L}$ = 1 pF). Comparing the experimental and finite element simulation results, it is observed that the NF-IBCC system’s gain displays high-pass characteristics in the frequency range of 1–10 MHz when $Z_{L}$ = 50 Ω. Conversely, when $Z_{L}=\frac{1}{sC_{L}}$ (with $C_{L}$ = 1 pF), the gain exhibits flat-band characteristics. These results are consistent with the descriptions provided by Equations (15) and (16). In addition, the results show that the measured gain exhibits a variation of within 2.8 dB.

Figure 16b provides the gain of the NF-IBCC system when $C_{L}$ is 1 pF, 15 pF, and 100 pF, respectively. From the experimental results, finite element simulation results, and the results described by Equation (16), it can be concluded that there is a negative correlation between the gain of the NF-IBCC system and the value of $C_{L}$. Specifically, the smaller the value of $C_{L}$, the greater the gain of the system. In addition, the results show that the measured gain exhibits a variation of within 2.9 dB.

In addition, Figure 16a,b reveal that when $Z_{L}$ = 50 Ω, the experimental and simulation results are in agreement with the description provided by Equation (15) in terms of magnitude. However, when $Z_{L}=\frac{1}{sC_{L}}$, Equation (16) does not accurately predict the magnitude of the NF-IBCC system’s gain. The study of power gain takes into account impedance matching considerations, unlike voltage gain. In the case where the load impedance ($Z_{L}$) is set to 50 Ω, the impedance of the source and load is symmetrical, and the impedance of the NF-IBCC channel is also nearly symmetrical. This symmetry results in the power gain being equal to the voltage gain. Hence, although Equation (15) implicitly accounts for impedance matching by representing voltage gain, when $Z_{L}=\frac{1}{sC_{L}}$, the symmetry of impedance is disrupted, causing the power gain to deviate from the voltage gain. However, Equation (16) still represents the voltage gain, neglecting the impedance matching issue. This discrepancy between Equation (16) and the other two cases leads to inconsistencies between the results obtained from Equation (16) and those derived from physical experiments and finite element simulations. Equation (16) can only provide a qualitative description of the passband characteristics and a negative correlation between the gain of the NF-IBCC system and the value of $C_{L}$.

## 4. Summary and Discussion

Compared to existing RF communication technologies, NF-IBCC offers the benefits of low power consumption and high information security. This makes it a promising communication technology to support healthcare systems by enabling the early detection and prevention of diseases through active health screening technology.

The existing research work has shown significant differences in the magnitude of the channel gain and the passband characteristics of the channel in NF-IBCC. To address this issue, this paper proposes a method that combines equivalent circuit models, finite element models, and physical experiments to analyze and verify the core parameters that determine the gain of the NF-IBCC system. The results reveal that the coupling capacitance, $C_{H}$ and $C_{air}$, and load impedance, $Z_{L}$, are critical parameters.

The series-equivalent capacitance of $C_{H}$ and $C_{air}$ primarily determines the channel gain of NF-IBCC, which is significantly impacted by the coupling capacitance with the smaller capacitance value between the two. The value of $C_{H}$ is determined primarily by weight, height, and distance, as demonstrated in fitting Equations (4) and (5). The accuracies of these equations were validated through physical experiments and finite element simulations. The value of $C_{air}$ was determined primarily by the physical size and distance of the transceiver grounds, as shown in fitting Equation (13). The accuracy of this equation was also verified through physical experiments and finite element simulations. Through analysis and verification, it was discovered that the value of $C_{air}$ is significantly smaller than that of $C_{H}$ at the same distance, making the channel gain of NF-IBCC heavily dependent on $C_{air}$. Therefore, in the process of studying NF-IBCC, the choice of measuring instruments is very important. It is necessary to choose a measuring instrument that is suitable for the actual application scenario, as much as possible, to provide the NF-IBCC system with an accurate channel gain. Hence, it is advisable to avoid using common-ground instruments, such as vector network analyzers, when measuring NF-IBCC. Such instruments short-circuit the return path of NF-IBCC and only measure the gain of the forward path, leading to results that are more optimistic than the actual values. Additionally, the ground of the measuring instrument should be consistent with the physical size of the ground of the transceiver in the NF-IBCC system. If the physical size of the ground of the measuring instrument is greater than the ground of the transceiver in the practical NF-IBCC system, it can also result in overestimated gain values.

On the other hand, the load impedance, which can either be resistance or capacitance, is an essential parameter that determines the passband characteristics of the NF-IBCC system. For instance, when the load impedance is $Z_{L}$ = 50 Ω, the gain of the NF-IBCC system exhibits a high-pass characteristic, whereas, when $Z_{L}=\frac{1}{sC_{L}}$, the gain becomes flat-band, indicating an increase in the gain of the low-frequency part. In addition, when $Z_{L}=\frac{1}{sC_{L}}$, the gain of the NF-IBCC system will increase as the value of $C_{L}$ decreases. Nevertheless, when $Z_{L}=\frac{1}{sC_{L}}$, analyzing the system requires the use of voltage gain, which may result in an impedance imbalance.

**Figure 17:**
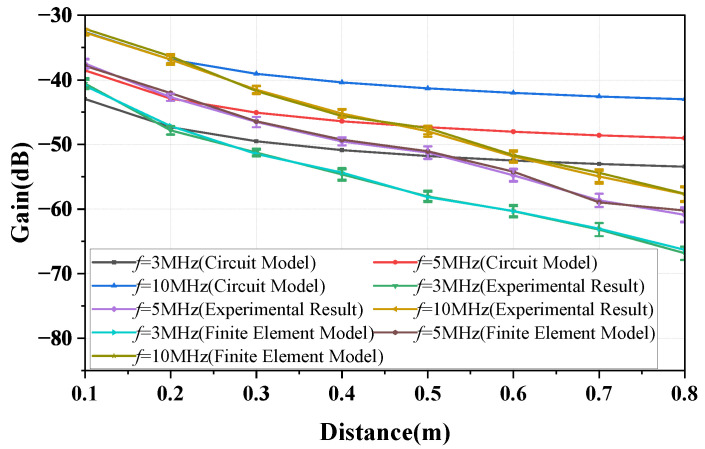
The variation curves of the NF-IBCC system gain with distance are obtained through a combination of simplified circuit model analysis, finite element simulation, and physical experiment measurements for load impedances of 50 ohms and frequencies of 3, 5, and 10 MHz.

This paper presents the model for the forward path, the return path, and the simplified model for the overall channel, where the return path 2 can be disregarded. Figure 17 presents the gain versus distance plots for frequencies of 3, 5, and 10 MHz. The curves represented by the *circuit model* in the figure are obtained based on the simplified equivalent circuit model (described by Equation (15)). The ’plots’ figure demonstrate that the results described by Equation (15) differ from the physical experiment results and finite element simulation results when the distance between the bodies is greater than 0.3 m. The discrepancy becomes increasingly significant as the distance increases. Therefore, it can be concluded that the applicable conditions of the simplified equivalent model are *d*≤ 0.3 m and $Z_{L}$ = 50 Ω. In addition, the results show that the measured gain exhibits a variation of within 2.6 dB.

However, the simplified equivalent circuit model can provide a qualitative understanding of the passband characteristics and the relationship between the gain of the NF-IBCC system and the value of $C_{L}$, but it cannot accurately predict the gain in this case due to the presence of impedance imbalance. Therefore, a more detailed analysis is needed to accurately predict the gain under this condition.

By analyzing and verifying the influence of core parameters, such as the coupling capacitance between two bodies, the coupling capacitance between the transceiver grounds, and the load impedance on the gain of the NF-IBCC system, this paper provides insights into why existing research shows large differences in channel characteristics. Moreover, the paper proposes a simplified equivalent circuit model for the overall channel that can predict the gain accurately for a limited range of distances and source/load impedances. The research conducted on the NF-IBCC system has provided important insight into the factors that affect the performance of the system and the development of theoretical models that can be used to optimize its performance. It is expected that the results of this research will help in the design and development of more efficient and reliable NF-IBCC systems.

## 5. Conclusions

By conducting equivalent circuit model analysis, finite element simulations, and physical experiments, we revealed the core parameters of the NF-IBCC system: $C_{H}$, $C_{air}$, and $Z_{L}$. Specifically, we found that the gain of the forward path of NF-IBCC is primarily dependent on $C_{H}$, whereas the gain of the return path mainly relies on $C_{air}$. Furthermore, the overall channel gain is mainly determined by the series-equivalent capacitance of $C_{H}$ and $C_{air}$, with $C_{air}$ having a slightly larger impact on the channel gain, despite its smaller capacitance. Additionally, the gain–passband behavior of NF-IBCC is primarily influenced by $Z_{L}$. Specifically, when $Z_{L}$ is set to 50 Ω, the gain exhibits high-pass characteristics, while setting $Z_{L}$ to $\frac{1}{sC_{L}}$ results in flat-band characteristics. These findings help to explain the substantial differences in the channel gain and passband characteristics observed in existing research on NF-IBCC. In addition, we developed a simplified equivalent circuit model that accurately captures the gain of the NF-IBCC system.

While our proposed simplified equivalent circuit model accurately captures the channel characteristics of the NF-IBCC system by extracting its core parameters, it is important to acknowledge that the model’s adaptation conditions are relatively stringent, with the requirements of $Z_{L}$ = 50 Ω and d≤ 0.3 m. These conditions may limit the model’s ability to accurately describe the channel characteristics under scenarios where the load impedance is $Z_{L}=\frac{1}{sC_{L}}$ or when the distance exceeds 0.3 m. This limitation could potentially restrict the application scope of NF-IBCC technology.

Therefore, in future work, we will address this limitation by developing a channel model that accommodates a broader range of conditions. This includes expanding the range of load impedances and extending the distance range for which the model can accurately describe the channel characteristics. By doing so, we aim to enhance the versatility and applicability of NF-IBCC technology in various scenarios.

## Data Availability

The data presented in this study are available on request from the corresponding author. The data are not publicly available due to privacy.
